# Chemical Oscillations With Sodium Perborate as Oxidant

**DOI:** 10.3389/fchem.2020.561788

**Published:** 2020-09-30

**Authors:** Krisztina Kurin-Csörgei, Eszter Poros-Tarcali, István Molnár, Miklós Orbán, István Szalai

**Affiliations:** Laboratory of Nonlinear Chemical Dynamics, Department of Analytical Chemistry, Institute of Chemistry, L. Eötvös University, Budapest, Hungary

**Keywords:** oscillatory reactions, perborate, non-linear dynamics, pH-oscillator, copper(II)-catalysis

## Abstract

The peroxo compounds H_2_O_2_ and K_2_S_2_O_8_ represent one of the major constituents in many oscillatory chemical systems. In this contribution we demonstrate that beside H_2_O_2_ and S_2_${\text{O}}_{8}{\;}^{2-}$ the sodium perborate (NaBO_3_·H_2_O) can act as alternative oxidizing agent in oscillatory reactions. So far the H_2_O_2_ has been successfully substituted with NaBO_3_ in two oscillators: in the BO${\;}_{3}^{-}$–S_2_${\text{O}}_{3}{\;}^{2-}$–Cu(II) flow system potential and pH oscillations, in the strongly alkaline Cu(II)-catalyzed BO${\;}_{3}^{-}$–SCN^−^ batch reaction, which are rather different in their chemistry and dynamics, potential oscillations were observed. In spite of the significant differences in the oxidizing nature of H_2_O_2_ and NaBO_3_ we assume that the oscillatory cycle in the BO${\;}_{3}^{-}$–substrate and in the H_2_O_2_–substrate systems is similar in many aspects, therefore the numbers of this new subgroup of the oscillators may be considered to be borate-mediated H_2_O_2_ oscillators. Mechanisms are suggested and simulations are shown to describe the oscillatory behaviors observed in the perborate chemistry based oscillators by using the assumption that the oxidation reactions of the intermediates (HO)_3_B(OOH)^−^ and (HO)_2_B(OOH)${\;}_{2}^{-}$ anions, which are dominant species in alkaline and neutral pH solutions of perborate, are much faster than that of H_2_O_2_.

## Introduction

The known oscillatory chemical reactions in which peroxo compounds are involved as oxidant include the classical Bray (H_2_O_2_–IO${\;}_{3}^{-}$) and Briggs-Rauscher [H_2_O_2_–IO${\;}_{3}^{-}$–MA—Mn(II)] reactions, some one-substrate pH-oscillators [H_2_O_2_–S^2−^; H_2_O_2_–S_2_${\text{O}}_{3}{\;}^{2-}$–Cu(II); H_2_O_2_–S_2_${\text{O}}_{4}{\;}^{2-}$; H_2_O_2_–Fe(CN)${\;}_{6}{\;}^{4-}$], a few two-substrate pH-oscillators [H_2_O_2_−${\text{SO}}_{3}{\;}^{2-}$–Fe(CN)${\;}_{6}{\;}^{4-}$; H_2_O_2_–${\text{SO}}_{3}{\;}^{2-}$–S_2_${\text{O}}_{3}{\;}^{2-}$; H_2_O_2_–HSO${\;}_{3}^{-}$–HCO${\;}_{3}^{-}$], the Cu(II) catalyzed H_2_O_2_–SCN^−^ and S_2_${\text{O}}_{8}{\;}^{2-}$–S_2_${\text{O}}_{3}{\;}^{2-}$ systems, and the Ag(I)-catalyzed S_2_${\text{O}}_{8}{\;}^{2-}$–S^2−^ reaction. Among the listed systems two representative oscillators, the Cu(II)-catalyzed H_2_O_2_–S_2_${\text{O}}_{3}{\;}^{2-}$ (Orbán and Epstein, 1987) and the H_2_O_2_–SCN^−^ reactions (Orbán, 1986) were selected to test for observing oscillations when the H_2_O_2_ is replaced with NaBO_3_. In the H_2_O_2_–S_2_${\text{O}}_{3}{\;}^{2-}$–Cu(II) system addition of acid (H_2_SO_4_), in the H_2_O_2_–SCN^−^–Cu(II) reaction addition of base (NaOH) as auxiliary reagent was also necessary to tune the systems to the oscillatory domain. Both oscillators are highly reproducible, they show wide variety of dynamical phenomena and the main reaction steps in their chemical mechanism are well-established. The H_2_O_2_–S_2_${\text{O}}_{3}{\;}^{2-}$–Cu(II) system represents a typical pH oscillator. In a CSTR it exhibits large amplitude pH and potential oscillations and bistability between high pH (~9) and low pH (~5) steady states in a broad range of chemical compositions. The H_2_O_2_–SCN^−^–Cu(II) reaction displays even richer dynamics. Under flow conditions it shows three types of bistability involving low and high potential steady states and an oscillatory state. In the oscillatory regime periodic changes appear in the potential of Pt and copper selective electrodes, in the color between yellow and colorless and in the rate of oxygen gas evolution, the pH is high (≥9) and steady. It is a unique feature of the H_2_O_2_–SCN^−^–Cu(II) system that it oscillates even at zero flow rate, i.e., at batch as well. During batch oscillations, the pH decreases slowly and smoothly. This oscillator has been used in many applications, for example, it is capable to generate oscillatory luminescence by adding luminol (Iranifam et al., 2010), it constituted the basis of analytical determination of many organic compounds present in a trace amount (<10^−8^ M) by following the changes in its oscillatory characteristics due to the interaction between the components of the oscillator and the target species (Jiménez-Prieto et al., 1997), it is also used for demonstration of chemical oscillations in classroom (Pimieta and Micheau, 1999).

The motivation of our work was to extend further the variety of the oscillatory chemical reactions by introducing such oxidant, sodium perborate, which has not been suggested so far to use as key component in giving rise to periodic changes in chemical systems. Beside seeking after non-linear systems, which are novel in composition, their background chemistry and simulation of their dynamical behavior are also subject to discussion in this study.

## Materials and Methods

The sodium perborate (NaBO_3_·H_2_O, Acros Organics), sodium thiosulfate (Na_2_S_2_O_3_·5H_2_O, Reanal), potassium thiocyanate (KSCN, VWR Chemicals), copper sulfate and nitrate (CuSO_4_·5H_2_O, Cu(NO_3_)_2_·3H_2_O, VWR Chemicals) were available commercially and used without further purification. The NaBO_3_ content of the solid reagent was analyzed by permanganometry, its purity was 95.0%. For the reaction mixtures used in the experiments four stock solutions were prepared: 0.1 M NaBO_3_·H_2_O, 0.1 M Na_2_S_2_O_3_·5H_2_O, 0.1 M KSCN, 4 × 10^−2^ M CuSO_4_·5H_2_O. The perborate stock solution was prepared daily. The aqueous solution of perborate undergoes slow decomposition, but its rate is slow and negligible during the time of an experiment, only 5% loss of the oxidation power of the stock solution was measured after 8 hours of its preparation. This stock solution is highly alkaline (pH > 10) due to the hydrolysis of NaBO_3_. The experiments have been carried out in a double wall glass reactor of *V* = 30 cm^3^, thermostated at *T* = 25°C, and operated under flow and batch configuration. The reaction vessel was equipped with combined glass electrode (Radelkis OP-0823P) and a combined Pt-calomel electrode (Mettler Toledo). In CSTR (abbreviation of Continuously-fed Stirred Tank Reactor) mode the reactor was fed with the appropriate reagents by a Gilson Minipuls peristaltic pump through four inlet tubes. The constant volume of the reactor content was ensured by removal of the outflow solution through the hole in the cup by another pump which rotated in the opposite direction. The stirring rate of 500 rpm was applied using a Heidolph MR 3000 stirrer. The electrodes were connected to pH-meters (Hanna pH-209). The responses of the electrodes were collected by personal computer through a pH-meter and a 16 bit A/D converter (NI-6010-PCI) with a sampling rate of 2 Hz. In the batch experiments the same experimental setup was used, here the reagents were mixed directly in the reactor dish.

The simulations were performed with the program XPPAUT [XPPAUT 8.0, 2016], by using cvode stiff solver, which based on a backward differentiation formula method. The step size, the absolute and the relative error tolerance were 10^−2^, 10^−15^, and 10^−5^, respectively. The general form of the differential equations used in the CSTR simulations was the following:

$$\begin{array}{l} \text{d}[{\text{X}}_{\text{i}}]/\text{dt}\;=\Sigma_{\text{j}}{\text{x}}_{\text{ij}}v_{\text{j}}+k_{0}({\left[{\text{X}}_{\text{i}}\right]}_{0}-\;[{\text{X}}_{\text{i}}]) \end{array}$$

where [X_i_] is the concentration of component X in the reactor, x_ij_ is the stoichiometric number of that species in the jth reaction, *k*_0_ is a first order rate constant corresponds to the inflow and outflow in a CSTR, and [X_i_]_0_ is the concentration of a component X in the input flow. The calculations of the equilibrium diagram were made by the program Medusa (Medusa).

## Results

Using the procedures described in the Materials and Methods section we were successful with observing periodic responses in both perborate—reductant—Cu(II) systems but the oscillatory concentrations differed significantly from those when H_2_O_2_ was used as oxidant. At this stage of our research we have not intended to establish the complete oscillatory domain and phase diagrams, our aim was just to find “optimum concentrations” and conditions where (and in the vicinity of them) the perborate oxidation takes place in oscillatory manner.

### BO${\;}_{3}^{-}$–S_2_O${\;}_{3}^{2-}$–Cu(II) Flow System

**Figure 1:**
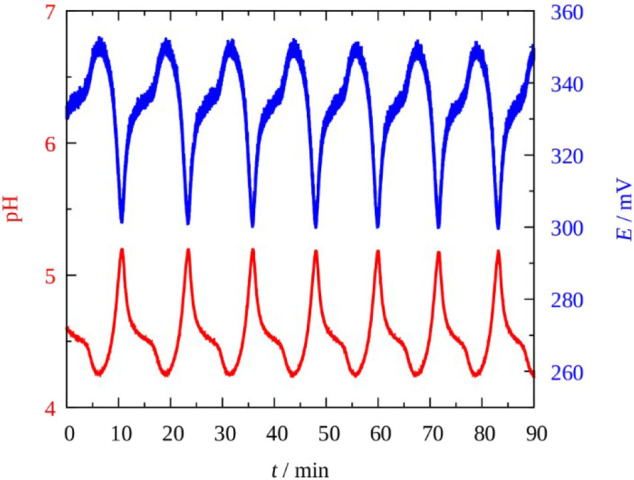
pH (red) and potential oscillations in the Pt vs. SCE electrode pair (blue) in the Cu^2+^-catalyzed sodium perborate–sodium thiosulfate CSTR system. [NaBO_3_]_0_ = 0.075 M; [H_2_SO_4_]_0_ = 0.0375 M; [Na_2_S_2_O_3_]_0_ = 5 × 10^−3^ M; [Cu^2+^]_0_ = 5 × 10^−5^ M; *k*_0_ = 2 × 10^−3^s^−1^; *T* = 25°C.

In the BO${\;}_{3}^{-}$–S_2_O${\;}_{3}^{2-}$–Cu(II) flow system—like in the H_2_O_2_ version—the bistability and oscillations in the pH and in the potential of the Pt vs. calomel electrode pair readily appeared. However, only much lower concentrations of perborate than that of H_2_O_2_ could be tested, due to the poor solubility of NaBO_3_·H_2_O (15 g/dm^3^ at *T* = 20°C). Besides, much higher input acid had to be introduced into the reaction mixture to partly neutralize the strong alkalinity originated from the hydrolysis of sodium perborate. The oscillatory potential and pH traces vs. time, measured in the perborate—thiosulfate—copper(II) system at an “optimum input concentration” are shown in Figure 1.

### BO${\;}_{3}^{-}$–SCN^−^–Cu(II) Batch System

**Figure 2:**
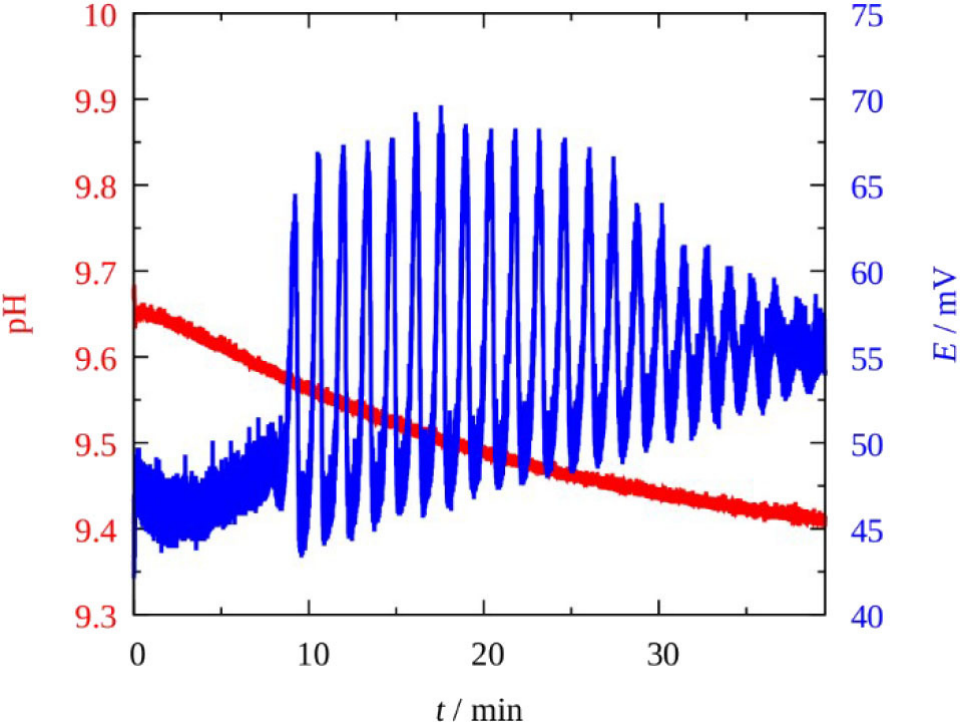
Potential oscillations in the Pt vs. SCE electrode pair (blue) and the change in the pH (red) in the Cu^2+^-catalyzed sodium perborate–potassium thiocyanate batch system. [NaBO_3_] = 0.030 M; [HCl] = 1.5 × 10^−3^ M; [KSCN] = 0.025 M; [Cu^2+^] = 10^−4^ M; *T* = 25°C.

**Figure 3:**
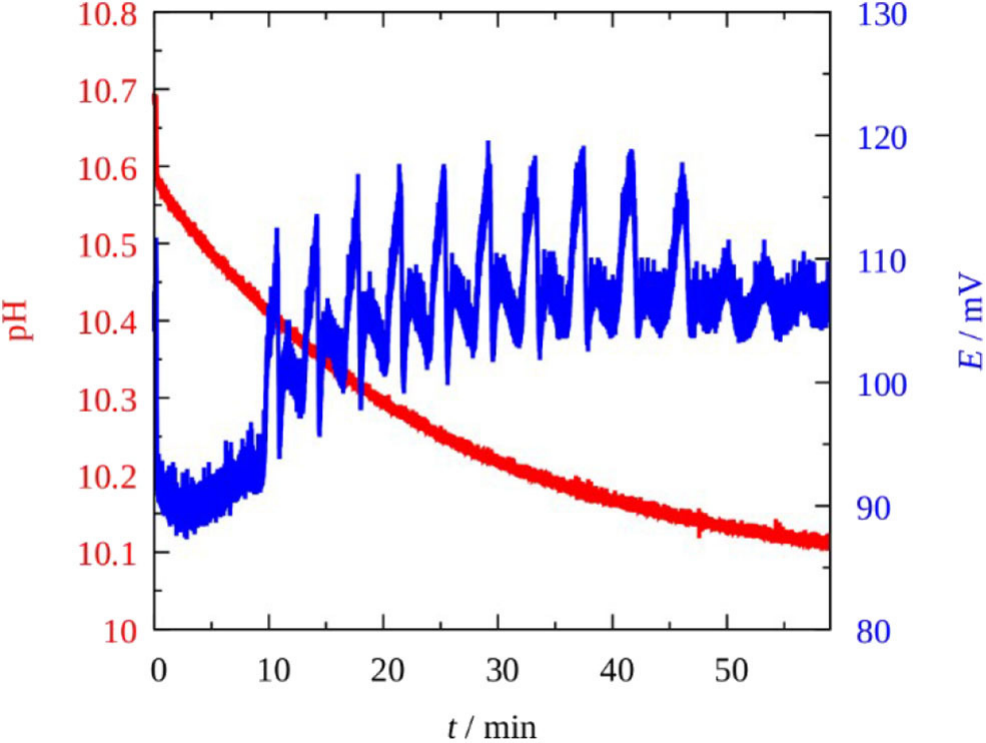
Complex potential oscillations in the Pt vs. SCE electrode pair (blue) and the change in the pH (red) in the Cu^2+^-catalyzed sodium perborate–potassium thiocyanate batch system. [NaBO_3_] = 0.010 M; [KSCN] = 0.025 M; [Cu^2+^] = 4 × 10^−4^ M; *T* = 25°C.

The BO${\;}_{3}^{-}$–SCN^−^–Cu(II) reaction behaved also in oscillatory fashion but it exhibited poorer dynamics compared to the original H_2_O_2_–SCN^−^–Cu(II) system. Up to now we failed to produce experimentally measured bistabilities or oscillations in CSTR, which were observed in the H_2_O_2_ version. However, our expectation, to find more examples where perborate plays the role of oxidant in oscillatory reaction was fulfilled: in the BO${\;}_{3}^{-}$–SCN^−^–Cu(II) reaction potential oscillations appeared but only under batch condition, and in a pH ≥ 9.5 solutions. The oscillations in the potential of a Pt electrode and the pH vs. time traces recorded under batch condition at an “optimum concentration” is shown in Figures 2, 3 shows complex type oscillations recorded at lower initial pH and higher [Cu(II)] than used in Figure 2. Within a range of concentrations, [NaBO_3_] ~ [KSCN] = 0.005–0.05 M, [CuSO_4_·5H_2_O] = 5 × 10^−5^–0.001 M, generally 10–20 cycles with amplitude of 15–20 mV and period of 5–10 min appear after an induction period of 5–10 min. The pH remains nearly constant (pH ≥ 9.5), it decreases a few tenth of a unit during a run. The perborate—thiocyanate—Cu(II) mixture is yellow (even without SCN^−^) due to the formation of copper-peroxo complexes [HO_2_-Cu(I) and/or HO_2_-Cu(OH)${\;}_{2}^{-}$] between the catalyst and superoxo radicals (HO${\;}_{2}^{\cdot }$, OH^·^, O${\;}_{2}^{\cdot -}$) which are involved in the mechanism of the peroxide oxidation reactions. The highly intense yellow color (absorption coefficient ε > 1,600 M^−1^cm^−1^ at λ = 345 nm) may cover up the color oscillations if there are any. The potential oscillations are accompanied with O_2_ evolution and with formation of a brown compound which is supposed to be a ring structured dimer of the copper peroxide (Luo et al., 1988). The reaction mixture becomes colorless when the oscillatory state terminates and the precipitate settles. A slow sigmoidal increase in the potential indicates that the reaction between BO${\;}_{3}^{-}$ and SCN^−^ still proceeds and by analogy of the reaction between H_2_O_2_ and SCN^−^ the following brutto stoichiometry is supposed to prevail (Wilson and Harris, 1961):

$$\begin{array}{l} 4{\text{BO}}_{3}^{-}+{\text{CN}}^{-}+3{\text{H}}_{2}\text{O}\to 4{\text{BO}}_{2}^{-}+{\text{HSO}}_{4}^{-}+{\text{HCO}}_{3}^{-}+{\text{NH}}_{4}^{+} \end{array}$$

Among the products (using copper nitrate as catalyst), the presence of SO${\;}_{4}^{2-}$ (with Ba^2+^) and that of NH${\;}_{4}^{+}$ (with Nessler reagent) could be detected.

## Numerical Simulations

**Figure 4:**
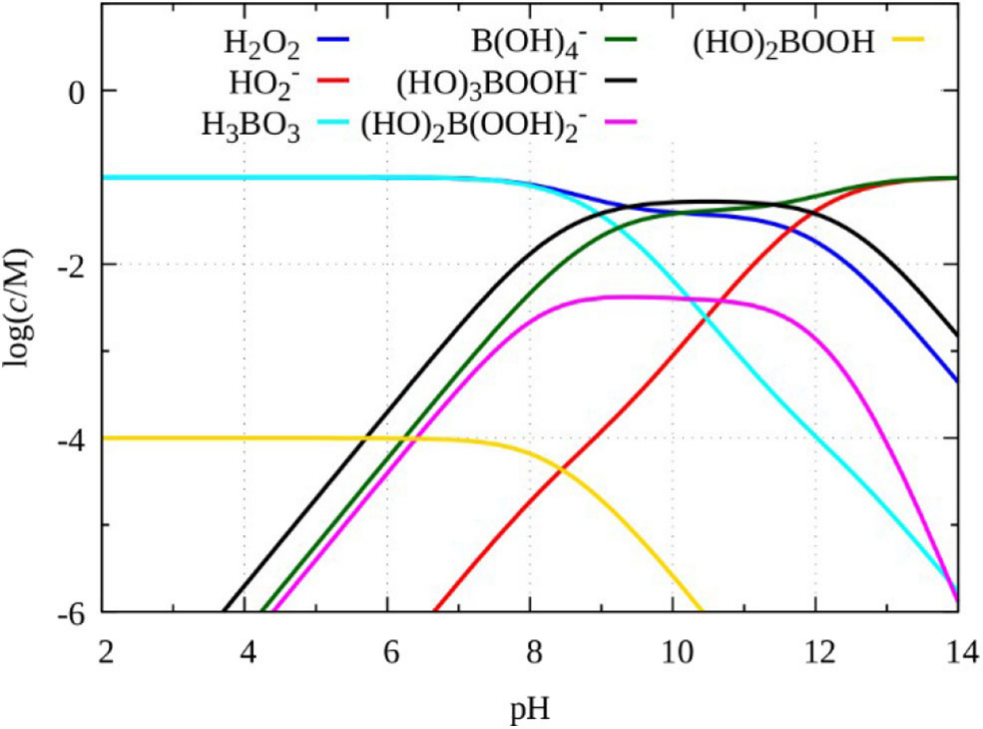
Species distribution in a mixture of 0.1 mol/dm^3^ boric acid and 0.1 mol/dm^3^ H_2_O_2_ calculated by using reactions R4, R5, R9, R10, R11, and R16.

To clarify the role of the perborate species in these oscillatory systems we performed numerical simulations. It is well-established that below pH = 7 perborate species are not stable, therefore in acid solution the active oxidant is hydrogen peroxide. Obviously, the mechanisms of these perborate oscillators must include the corresponding hydrogen peroxide chemistry. The question, what we address here is the following: do the perborate species contribute substantially to the development of the oscillations or the dynamics is mainly governed by the reactions of hydrogen peroxide? According to the literature between pH = 8 and 12 the kinetic effect of the existing different perborate species can be significant (Burgess and Hubbard, 2013). Independent studies indicate that (HO)_2_B(OOH) is unreactive while the analogous reactions of (HO)_3_B(OOH)^−^ and (HO)_2_B(OOH)${\;}_{2}^{-}$ anions are much faster than that of the H_2_O_2_ (Davies et al., 2005; Lobachev et al., 2016). The equilibrium distribution of the different perborate species in a perborate solution, which in acidic medium is a 1:1 mixture of H_3_BO_3_ and H_2_O_2_, as function of pH (Figure 4) was calculated by following the paper of Pizer and Tihal (1987). In the calculation all relevant equilibrium processes, R4, R5, R9, R10, R11 reactions (Tables 1, 2), and the formation of (HO)_2_B(OOH) in reaction R16 (Pizer and Tihal, 1987), were considered.

$$\begin{array}{l} {\text{H}}_{3}{\text{BO}}_{3}+{\text{H}}_{2}{\text{O}}_{2}⇌{\left(\text{HO}\right)}_{2}\text{B}(\text{OOH})+{\text{H}}_{2}{\text{OK}}_{16}=\;0.01{\text{M}}^{-1}\;\text{R}16 \end{array}$$

The result clearly shows that in the range of pH = 8–12 (HO)_3_B(OOH)^−^ is the dominant form of perborate but (HO)_2_B(OOH)${\;}_{2}^{-}$ also exists in a relatively high ratio. As the concentration of (HO)_3_B(OOH)^−^ in this pH range is larger than that of the hydrogen peroxide, it is likely that its kinetic role is not negligible.

**Table 1:** Model of the H_2_O_2_–S_2_O${\;}_{3}^{2-}$–Cu(II) system.

**Step**	**Reaction**	**Reaction rate**	**Rate constant**
R1	H_2_O_2_ + S_2_O${\;}_{3}^{2-}$ → HOS_2_O${\;}_{3}^{-}$+ OH^−^	*v*_1_ = *k*_1_[H_2_O_2_][S_2_${\text{O}}_{3}{\;}^{2-}$] + *k*_1_′[H_2_O_2_][S_2_${\text{O}}_{3}{\;}^{2-}$][OH^−^][Cu^2+^]_T_	*k*_1_ = 0.019 M^−1^ s^−1^ *k*_1_′ = 3·10^8^ M^−3^ s^−1^
R2	3H_2_O_2_ + HOS_2_O${\;}_{3}^{-}$ → 2SO${\;}_{4}^{2-}$+ 3H^+^ + 2H_2_O	*v*_2_ = *k*_2_[H_2_O_2_][HOS_2_O${\;}_{3}^{-}$]	*k_2_* = 0.6 M^−1^ s^−1^
R3	S_2_O${\;}_{3}^{2-}$+ HOS_2_O${\;}_{3}^{-}$ → S_4_O${\;}_{6}^{2-}$+ OH^−^	*v*_3_ = *k*_3_[S_2_${\text{O}}_{3}{\;}^{2-}$][HOS_2_O${\;}_{3}^{-}$]	*k*_3_ = 60 M^−1^ s^−1^
R4	H_2_O ⇌ OH^−^+ H^+^	*v_4_* = *k_4_*[H_2_O] – *k*_−4_[OH^−^][H^+^]	*k_4_*[H_2_O] = 10^−3^ M s^−1^ *k*_−4_ = 1.8·10^11^ M^−1^ s^−1^
R5	H_2_O_2_ ⇌ HO${\;}_{2}^{-}$+ H^+^	*v_5_* = *k_5_*[H_2_O_2_] – *k*_−5_[HO${\;}_{2}^{-}$][H^+^]	*k_5_* = 2.2·10^−2^ s^−1^ *k*_−5_ = 1.6·10^10^ M^−1^ s^−1^
R6	H_2_O_2_ + 2Cu(S_2_O_3_)${\;}_{2}^{3-}$+ 2H^+^ → 2Cu(S_2_O_3_)${\;}_{2}^{2-}+$ 2H_2_O	*v_6_* = *k_6_*[H_2_O_2_][Cu(S_2_O_3_)${\;}_{2}^{3-}$][H^+^]^2^	*k_6_* = 10^14^ M^−1^ s^−1^
R7	S_2_O${\;}_{3}^{2-}$+ Cu(S_2_O_3_)${\;}_{2}^{2-}$ → Cu(S_2_O_3_)${\;}_{2}^{3-}+$ S_2_O${\;}_{3}^{-}$	*v_7_* = *k_7_*[S_2_${\text{O}}_{3}{\;}^{2-}$][Cu(S_2_O_3_)${\;}_{2}^{2-}$]	*k_7_* = 10^10^ M^−1^ s^−1^
R8	2S_2_O${\;}_{3}^{-}$ → S_4_O${\;}_{6}^{2-}$	*v_8_* = *k_8_*[S_2_O${\;}_{3}^{-}$]^2^	*k_8_* = 10^4^ M^−1^ s^−1^

*Here [Cu^2+^]_T_ = [Cu(S_2_O_3_)${\;}_{2}^{3-}$] + [Cu(S_2_O_3_)${\;}_{2}^{2-}$]*.

**Table 2:** Reactions of the boron species related to the H_2_O_2_–S_2_O${\;}_{3}^{2-}$–Cu(II) system.

**Step**	**Reaction**	**Reaction rate**	**Rate constant**
R9	H_3_BO_3_ + H_2_O ⇌ B(OH)${\;}_{4}^{-}$+ H^+^	*v_9_* = *k_9_*[H_3_BO_3_] – *k*_−9_[B(OH)${\;}_{4}^{-}$][H^+^]	*k_9_*= 0.575 s^−1^ *k_−9_* = 10^9^ M^−1^ s^−1^
R10	H_3_BO_3_ + H_2_O_2_ ⇌ (HO)_3_B(OOH)^−^+ H^+^	*v_10_*= *k_10_*[H_3_BO_3_][H_2_O_2_] – *k*_−10_[(HO)_3_B(OOH)^−^][H^+^]	*k_10_*= 0.20 M^−1^ s^−1^ *k_−10_* = 10^9^ M^−1^ s^−1^
R11	(HO)_3_B(OOH)^−^+ H_2_O_2_ ⇌ (HO)_2_B(OOH)${\;}_{2}^{-}$+ H_2_O	*v_11_* = *k_11_*[(HO)_3_B(OOH)^−^][H_2_O_2_] – k_−11_[(HO)_2_B(OOH)${\;}_{2}^{-}$]	*k_11_*= 2·10^9^ M^−1^ s^−1^ *k_−11_* = 10^9^ s^−1^
R12	(HO)_3_B(OOH)^−^+ S_2_O${\;}_{3}^{2-}$+ H_2_O = HOS_2_O${\;}_{3}^{-}$+ 2OH^−^+ H_3_BO_3_	*v*_12_ = *f_*cat*_k*_1_[(HO)_3_B(OOH)^−^][S_2_${\text{O}}_{3}{\;}^{2-}$] + *f_*cat*_k*_1_′[(HO)_3_B(OOH)^−^][S_2_${\text{O}}_{3}{\;}^{2-}$][OH^−^][Cu^2+^]_T_	*f_*cat*_* adjustable
R13	3(HO)_3_B(OOH)^−^+ HOS_2_O${\;}_{3}^{-}$ = 2SO${\;}_{4}^{2-}$+ 3H_3_BO_3_ + 2H_2_O	*v_13_* = *f_*cat*_k*_2_[(HO)_3_B(OOH)^−^][HOS_2_O${\;}_{3}^{-}$]	*f_*cat*_* adjustable
R14	(HO)_2_B(OOH)${\;}_{2}^{-}$+ S_2_O${\;}_{3}^{2-}$+ H_2_O = HOS_2_O${\;}_{3}^{-}$+ OH^−^+ (HO)_3_B(OOH)^−^	*v*_14_ = *f_*cat*_k*_1_[(HO)_2_B(OOH)${\;}_{2}^{-}$][S_2_${\text{O}}_{3}{\;}^{2-}$] + *f_*cat*_k*_1_′[(HO)_2_B(OOH)${\;}_{2}^{-}$][S_2_${\text{O}}_{3}{\;}^{2-}$][OH^−^][Cu^2+^]_T_	*f_*cat*2_* adjustable
R15	3(HO)_2_B(OOH)${\;}_{2}^{-}$+ HOS_2_O${\;}_{3}^{-}$+ H_2_O = 2SO${\;}_{4}^{2-}$+ 3(HO)_3_B(OOH)^−^+ 3H^+^	*v_15_* = *f_*cat*_k*_2_[(HO)_2_B(OOH)${\;}_{2}^{-}$][HOS_2_O${\;}_{3}^{-}$]	*f_*cat*_* adjustable

*Here k_1_, k_1_′,k_2_ refer to Table 1*.

### BO${\;}_{3}^{-}$–S_2_O${\;}_{3}^{2-}$–Cu(II) Flow System

As a starting point we used the model of the H_2_O_2_–S_2_O${\;}_{3}^{2-}$–Cu(II) flow system published by Jedrusiak et al. (2017) recently, which an extended version of the original mechanism proposed by Kurin-Csörgei et al. (1996). This model consists of 8 reactions and 9 variables (Table 1) which is capable of simulating not only the pH oscillations but the time evolution of the concentration of the copper(II) and copper(I) species in the form of thiosulfate complexes. We extended this model with the formation and the oxidation reactions of (HO)_3_B(OOH)^−^ and (HO)_2_B(OOH)${\;}_{2}^{-}$ (Table 2). Reactions R12–R15 describe the catalytic effect of the perborate species on reactions R1 and R2, but at the moment we have no direct kinetic information about these reactions. As a first assumption we applied similar rate laws for these catalytic reactions like that of R1 and R2, but the rate constants are multiplied by a dimensionless factor *f*_*cat*_. We also assume that the reactivity of (HO)_3_B(OOH)^−^ and (HO)_2_B(OOH)${\;}_{2}^{-}$ are the same, therefore we apply identical rate constants for reactions R12 and R14.

**Figure 5:**
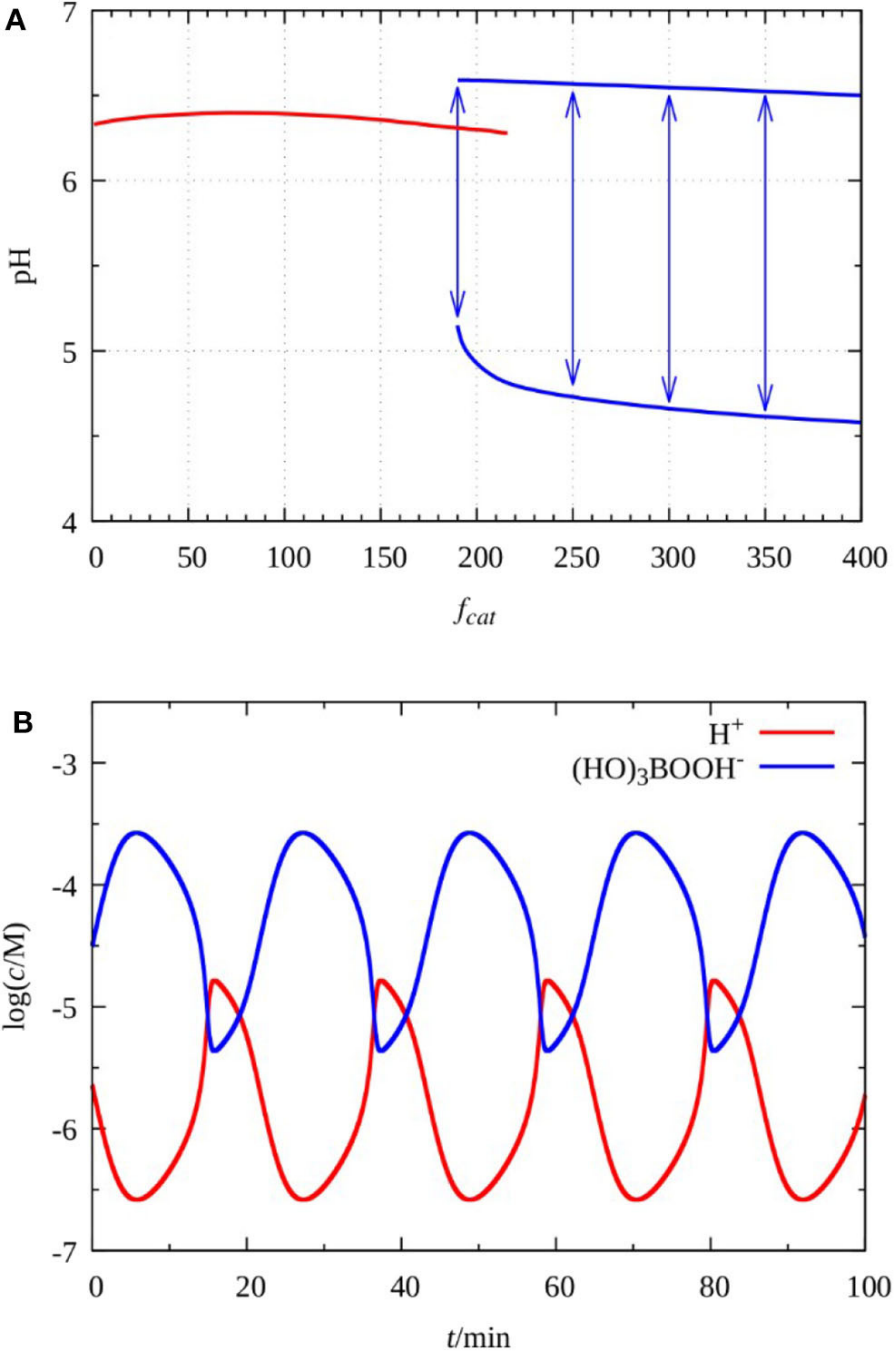
Simulated bifurcations diagram as a function of parameter *f*_*cat*_. The arrows indicates the amplitude of oscillations **(A)** and oscillations in the concentration of H^+^ and (HO)_3_B(OOH)^−^ at *f*_*cat*_= 220 **(B)**. The parameters of the simulations *k*_0_ = 0.002 s^−1^, [S_2_${\text{O}}_{3}{\;}^{2-}$]_0_ = 0.01 M, [(HO)_3_B(OOH)^−^]_0_ = 0.062 M, [Cu^2+^]_T_ = 1.5 × 10^−5^ M, [H^+^]_0_ = 0.066 M.

To clarify the potential role of (HO)_3_B(OOH) ^−^ and (HO)_2_B(OOH)${\;}_{2}^{-}$ in the dynamics of the system, we calculated a bifurcation diagram by varying the value of *f*_*cat*_. As it is presented in Figure 5A oscillations develop above a critical value of *f*_*cat*_, that is about 200, with a finite amplitude through a subcritical bifurcation. It is important to point out that at *f*_*cat*_= 0, that means hydrogen peroxide solely governs the system, the model does not show oscillations, at the conditions corresponding to the experiments presented here. This result confirms not only that the catalytic effect of the perborate species has a significant contribution in the development of oscillations, but this effect must be large enough. Typical concentration oscillations of H^+^ and (HO)_3_B(OOH) ^−^ are shown in Figure 5B. The amplitude in pH and the period is in good agreement with the experimental observations.

### BO${\;}_{3}^{-}$–SCN^−^–Cu(II) Batch System

A detailed mechanism of the H_2_O_2_–SCN^−^–Cu(II) oscillatory reaction was published by Luo et al. (1989), which consists of 30 reactions and 26 variables and many estimated rate constants. The extension of this model with the assumed reactions of the perborate species would result in a poorly defined mechanism, since we have no direct kinetic data about these reactions. To obtain information about the effect of the replacement of H_2_O_2_ by perborate, we decided to use a recently published simple five variables core model (Table 3), which was deduced from the original one by Wiśniewski et al. (2015). In this model the concentration of the oxidant, that is H_2_O_2_, is a constant parameter. When we apply this model for the perborate oscillator, we assume that there are at least two oxidants, hydrogen peroxide and one of the perborate species.

**Table 3:** The five variables model of the H_2_O_2_–SCN^−^–Cu(II) system (Wiśniewski et al., 2015).

**Step**	**Reaction**	**Reaction rate**	**Rate constant**
RI	*2H_2_O_2_* + *SCN^−^*→**OS(O)CN**^**−**^+ *2H_2_O*	*v_*I*_* = *k*_I_[*H_2_O_2_*][*SCN^−^*]	*k*_I_ = 1.33·10^−7^ M^−1^ s^−1^
RII	*OOS(O)CN^−^***+** **OS(O)CN**^**−**^+ *H_2_O* ⇌ **2OS(O)CN**^·^+ *2OH^−^*	*v*_II_ = *k*_II_[**OS(O)CN**^**−**^] – k_−*II*_[**OS(O)CN**^·^]^2^	*k_*II*_* = 5·10^−2^ s^−1^ *k*_−*II*_ = 4·10^6^ M^−1^ s^−1^
RIII	**Cu(SCN)**${\;}_{2}^{-}$+ **OS(O)CN**^·^+ *HO_2_*^·^+ 2*OH* ^·^→**OS(O)CN** ^**−**^+ **HO**_**2**_**Cu(OH)**${\;}_{2}^{-}$+ 2*SCN^−^*	*v*_III_ = *k*_III_[**Cu(SCN)**${\;}_{2}^{-}$][**OS(O)CN**^·^]	*k*_III_ = 1·10^6^ M^−1^ s^−1^
RIV	**HO**_**2**_**Cu(OH)**${\;}_{2}^{-}$+ 2*SCN^−^*→**Cu(SCN)**${\;}_{2}^{-}$+ **HO**_**2**_^·^+ 2*OH* ^·^	*v_*IV*_* = *k_*IV*_*[**HO**_**2**_**Cu(OH)**${\;}_{2}^{-}$]	*k_*IV*_* = 6·10^3^ M^−1^ s^−1^
RV	**2HO**_**2**_^·^→*H_2_O_2_* + *O_2_*	*v_*V*_* = *k_*V*_*[**HO**_**2**_^·^]^2^	*k_*V*_* = 2·10^2^ M^−1^ s^−1^

*The variables are: **OS(O)CN**^−^, **Cu(SCN)**_**2**_^−^, **OS(O)CN**^·^, **HO**_**2**_**Cu(OH)**_**2**_^−^, **HO**_**2**_^·^. The concentrations of the other reactants (H_2_O_2_, SCN^−^, H_2_O, OOS(O)CN^−^, OH^−^, HO${\;}_{2}^{-}$) are constant. The initial concentrations used in the simulations are: [H_2_O_2_][SCN^−^] = 0.075 M, [**OS(O)CN**^−^] = 10^−8^ M, [**Cu(SCN)**_**2**_^−^] = 10^−4^ M, [**OS(O)CN**^·^] = 10^−8^ M, [**HO**_**2**_**Cu(OH)**_**2**_^−^] = 10^−8^ M, [**HO**_**2**_^·^] = 10^−8^ M*.

According to the experiments there are some significant differences between the applied conditions in the H_2_O_2_–SCN^−^–Cu(II) and in the HBO_3_–SCN^−^–Cu(II) systems, which are the followings: (1) while the typical concentration of H_2_O_2_ is in the range of 0.1–0.5 M (Orbán, 1986), that of the perborate is in between 0.005 and 0.06 M; (2) the pH of the reacting medium is in between 10 and 11 in case of H_2_O_2_ but it is rather between 9 and 10 in the perborate system.

**Figure 6:**
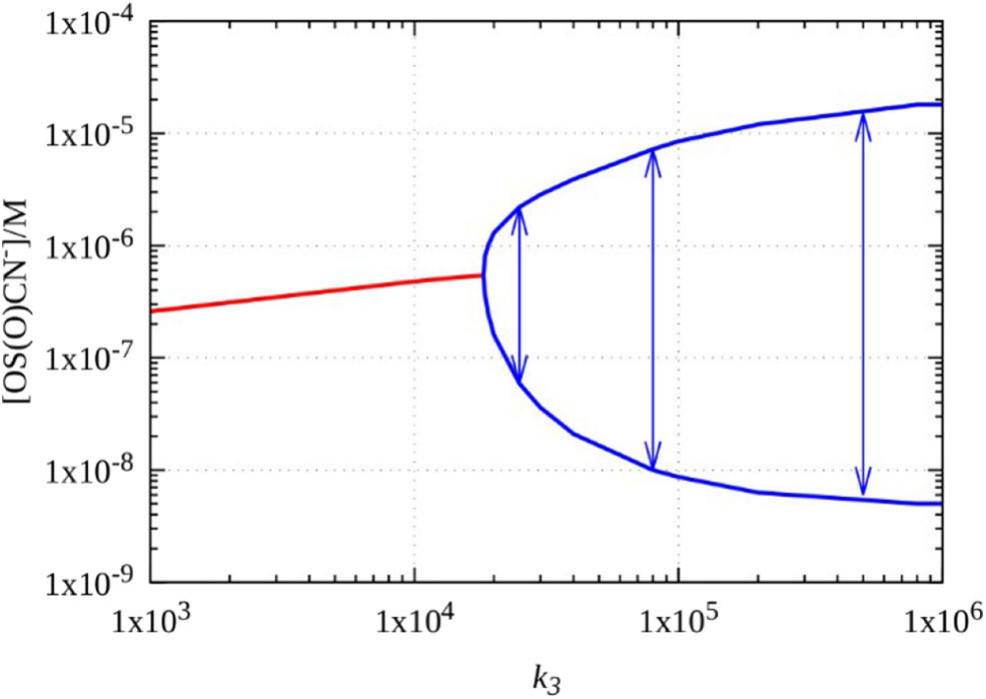
Simulated bifurcations diagram of the five variables model of the H_2_O_2_–SCN^−^–Cu(II) reaction as a function of parameter *k*_*III*_, the arrows indicates the amplitude of oscillations.

**Figure 7:**
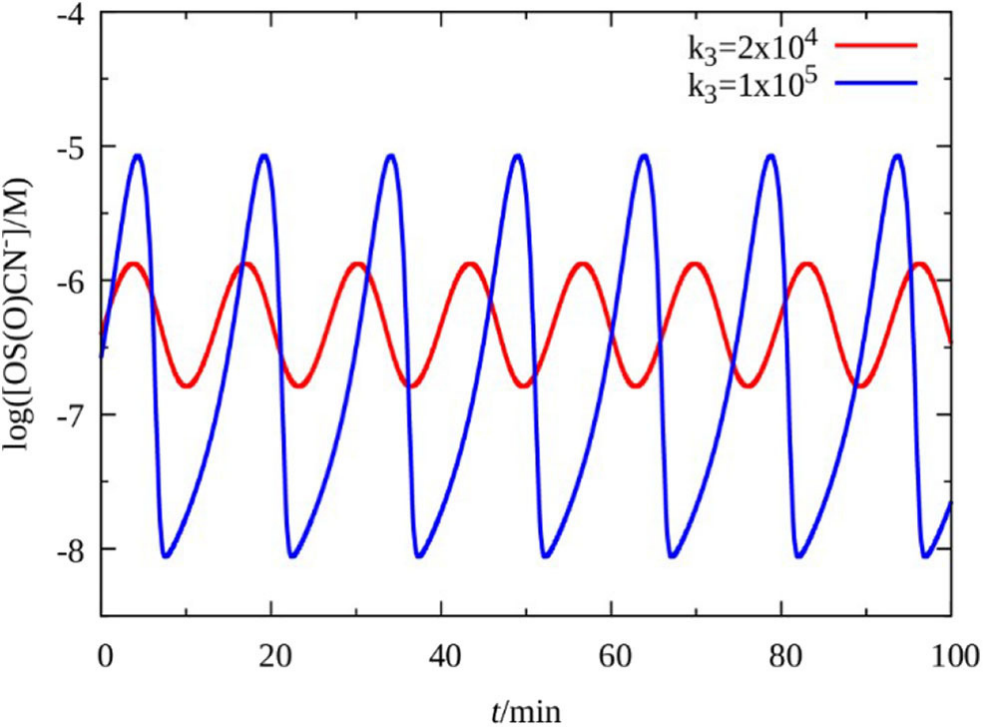
Simulated oscillations in the five variables model of the H_2_O_2_–SCN^−^–Cu(II) reaction as a function of parameter *k*_3_.

In the model the concentration of oxidant affects the rates of reactions RI and RIII. We found that, the dynamics of the model is quite insensitive to the variation of the rate of RI, as oscillations can be found at very low concentration of the oxidant, e.g., at 10^−6^ M. Therefore, we focused on reactions R3, where the oxidant in the original model is HO${\;}_{2}^{-}$, which has relatively high concentration, e.g., 0.03–0.04 M, at the usual conditions of the original H_2_O_2_–SCN^−^–Cu(II) oscillatory reaction. However, when perborate is applied at the lower pH as it is in the experiments, the estimated concentration of HO${\;}_{2}^{-}$ significantly lowers. Equilibrium calculations with total perborate concentration 0.005–0.06 M by using R4, R5, R9, R10, R11, and R16 show that concentration of HO${\;}_{2}^{-}$ drops to 10^−4^ M or even below. Consequently, the rate constant of RIII must be also decreased by the same factor, that is about 200, if only HO${\;}_{2}^{-}$ is considered as effective oxidant in this process. The value of k_III_ which fits to the actual concentration of HO${\;}_{2}^{-}$ is around 5·10^3^ M^−1^ s^−1^. Figure 6 represents a bifurcation diagram as the value of k_III_ is varied. The oscillations (Figure 7) appear through a supercritical Hopf bifurcations at k_III_ = 1.84·10^4^ M^−1^ s^−1^, that is 50 times smaller than the value expected for the case when solely HO${\;}_{2}^{-}$ is the oxidant. We concluded that in the perborate system HO${\;}_{2}^{-}$ itself as an oxidant cannot support the development of oscillations, because process RIII would be too slow. Therefore, we assume that the different perborate species [(HO)_3_B(OOH)^−^ and (HO)_2_B(OOH)${\;}_{2}^{-}$] are involved as oxidant in this process. The role of the perborate species in this compound reaction is likely to react with Cu(OH)_2_ to produce Cu(OH)_3_ and HO_2_Cu(OH)${\;}_{2}^{-}$.

## Discussion

The question may be raised whether the oscillations that appear in the perborate oxidations represent a new subgroup among the known chemical periodic reactions or if they are simply H_2_O_2_-based oscillators assisted by borate chemistry. To approach this question the species, their reactivity and the equilibria in which they are involved should be considered. Deary et al. (2013) listed more than a dozen of species and equilibria that may exist in a mixture of H_2_O_2_ and boric acid at different pHs. The general chemical formula of sodium perborate is NaBO_3_·nH_2_O (*n* = 1, 2, 4). It is often presumed to be a stabilized form of H_2_O_2_ because it functions like H_2_O_2_ does in many practical applications. However, the sodium perborate is not just an adduct of H_2_O_2_ with tetraborate like e.g., sodium percarbonate (2 Na_2_CO_3_·3 H_2_O_2_) or urea peroxide (CO(NH_2_)_2_·H_2_O_2_). In its aqueous solution, which is highly alkaline (pH ~ 11) the perborate is present in a form of dimer anion B_2_O_4_(OH)${\;}_{4}^{2-}$ (diperoxoborate) in which the two boron atoms are bound by two peroxide groups. In the perborate oxidation reactions, the diperoxo form releases H_2_O_2_ and B(OH)${\;}_{4}^{-}$ or H_3_BO_3_ and depending on the pH different species (shown already in Figure 4) play the key role in the oxidation process. At the high pH range (pH = 8–11) the reactive (HO)_2_B(OOH)${\;}_{2}^{-}$ and (HO)_3_B(OOH)^−^ anions are the major oxidant. At medium pHs the peroxoborates exist in equilibrium with H_2_O_2_, B(OH)${\;}_{4}^{-}$ or H_3_BO_3_,

$$\begin{array}{l} {\left(\text{HO}\right)}_{3}\text{B}{\left(\text{OOH}\right)}^{-}+{\text{H}}_{2}\text{O}↔{\text{H}}_{2}{\text{O}}_{2}+\text{B}{\left(\text{OH}\right)}_{4}^{-}\;\text{or}\\ {\left(\text{HO}\right)}_{3}\text{B}{\left(\text{OOH}\right)}^{-}+{\text{H}}_{2}{\text{O}}_{2}↔{\text{H}}_{2}{\text{O}}_{2}+{\text{H}}_{3}{\text{BO}}_{3}+\;{\text{OH}}^{-} \end{array}$$

here the perborates oxidize along with H_2_O_2_. In the acidic pH-range the equilibrium is completely shifted toward the formation of H_2_O_2_ and H_3_BO_3_ and the perborate reacts like H_2_O_2_. One of our plans is to use perborate in acidic environment. Here the perborate would function as reductant, like the H_2_O_2_ acts in the KMnO_4_–H_2_O_2_–H_3_PO_4_ oscillatory reaction (Nagy and Treindl, 1989).

A chemical background that prevails in the perborate oscillators is assumed to resemble pretty much to the processes which dominate in the analogous H_2_O_2_–S_2_${\text{O}}_{3}{\;}^{2-}$/SCN^−^–Cu(II) oscillators. The role and effect of the copper (II) catalyst is obviously similar in each system: the Cu(II) oxidizes the sulfur component and it is reduced to copper(I), what is temporarily stabilized by the reductant or its intermediate, then the Cu(I), being unstable in this oxidation state, is reoxidized to Cu(II) by the oxidant. Adding ligand which forms complex with copper ions influences this catalytic cycle. EDTA prevents to bring about oscillations in a mixture of oscillatory composition due to its high affinity to bind Cu(II) in a stable complex. In presence of NH_4_OH, however, when the rate of reduction of copper (II) is much slower than in its absence (Byerley et al., 1973), the duration of the oscillatory phase and the number of oscillations significantly increased. Exclusive role of Cu^2+^ as catalyst is shown by the fact, that when it was replaced with Ag^+^, Ni^2+^, Co^2+^, or Fe^2+^ oscillations were not observed.

As far as the mechanistic description is concerned most steps may be identical in the H_2_O_2_ and BO${\;}_{3}^{-}$ oscillators. The analogous initial steps in the oxidation of S_2_O${\;}_{3}^{2-}$ or SCN^−^ by H_2_O_2_ and BO${\;}_{3}^{-}$ are expressed in Equations (1)–(4):

(1) $$\begin{array}{lll} {\text{H}}_{2}{\text{O}}_{2}+{\text{S}}_{2}{\text{O}}_{3}^{2-} & \to & \text{HO}{\text{S}}_{2}{\text{O}}_{3}^{-}+\text{O}{\text{H}}^{-} \end{array}$$

(2) $$\begin{array}{l} {\text{BO}}_{3}^{-}+{\text{S}}_{2}{\text{O}}_{3}^{2-}+{\text{H}}_{2}\text{O }\to {\text{ HOS}}_{\text{2}}{\text{O}}_{3}^{-}+{\text{BO}}_{2}^{-}+{\text{OH}}^{-} \end{array}$$

(3) $$\begin{array}{lll} {\text{H}}_{2}{\text{O}}_{2}+\text{SC}{\text{N}}^{-} & \to & \text{OSC}{\text{N}}^{-}+{\text{H}}_{2}\text{O} \end{array}$$

(4) $$\begin{array}{l} {\text{BO}}_{\text{3}}^{-}\text{+ }{\text{SCN}}^{-}\;\to {\text{ OSCN}}^{-}\text{+ }{\text{BO}}_{\text{2}}^{-} \end{array}$$

In the formation of HOS_2_O${\;}_{3}^{-}$ and OSCN^−^ in Equations (2) and (4) a monocyclic three membered peroxide intermediate, called dioxaborirane [(HO)_2_BOO^−^] which forms from (HO)_3_B(OOH)^−^ by losing one H_2_O was proposed to act as most reactive species (Deary et al., 2013):


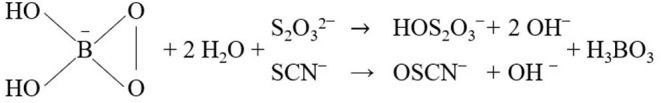


Further steps in the oxidation of HOS_2_O${\;}_{3}^{-}$ and OSCN^−^ intermediates are assessed to proceed through the reactions listed in Table 1, R1—R8 and in Table 3, RI—RV. In the systems where the reductant is S_2_O${\;}_{3}^{2-}$, the intermediate HOS_2_O${\;}_{3}^{-}$ formed in steps (1) or (2) undergoes two-way reaction (catalyzed by Cu(II)(S_2_O_3_)${\;}_{2}^{2-}$/Cu(I)(S_2_O_3_)${\;}_{2}^{3-}$) according to R2 and R3 in Table 1. Step R2 produces, while step R3 consumes H^+^ with a time-lag between them which results in oscillations in pH. When the substrate in the oscillators is SCN^−^, the oxidation of OSCN^−^ formed in Equation (3) or (4) takes place through reactions RI—RV in Table 3, The positive feedback, which one of the preconditions for oscillations to occur, is involved in RII + RIII.

The reactions of the boron species related to the H_2_O_2_–S_2_O${\;}_{3}^{2-}$–Cu(II) oscillatory systems are shown in Table 2, where the participation of the dominant boron intermediate (HO)_3_B(OOH)^−^ and its role in the oscillatory mechanism are clearly demonstrated.

## Conclusions

It is customary to categorize the known chemical oscillatory reactions into groups or families. The groups are usually named after the major component which is present in each member and which plays key role in bringing about periodic behavior in its reactions with appropriate partners. Up to now we have distinguished BrO${\;}_{3}^{-}$, BrO${\;}_{2}^{-}$, ClO${\;}_{2}^{-}$, IO${\;}_{3}^{-}$, IO${\;}_{4}^{-}$, MnO${\;}_{4}^{-}$, S_2_O${\;}_{8}^{2-}$, and H_2_O_2_-based oscillators with many variants in each class. All listed components function as powerful oxidant and each contains an element that possesses multiple oxidation states.

In this report we have demonstrated that sodium perborate (NaBO_3_·H_2_O), similarly to some oxyhalogens, permanganate or peroxides, can also be used as major reactant in constructing oscillatory reactions. It was proven by the successful replacement of H_2_O_2_ with NaBO_3_ which resulted in the extension of the number of chemical oscillators with two members: the BO${\;}_{3}^{-}$–S_2_O${\;}_{3}^{2-}$–Cu(II) and the BO${\;}_{3}^{-}$–SCN^−^–Cu(II) systems. Our associated experimental and numerical results show that this is not a simple replacement of the oxidant, but the development of oscillations is supported by the catalytic effect of borate on peroxide chemistry. pH oscillators are often applied as a source of periodic driving force to initiate rhythmic changes, motions or deformations in different physico-chemical systems. A new variant, like the BO${\;}_{3}^{-}$–S_2_O${\;}_{3}^{2-}$–Cu(II) system, may open some new possibilities in this direction. To approach this goal we intend to improve the perborate-based oscillators. Attempts will be made to find experimental conditions under which pH-oscillations of higher amplitude than were measured earlier, would appear. However, use of higher initial concentrations to enhance the pH-oscillations is hampered by the low solubility of NaBO_3_·H_2_O. The solubility problem may be to overcome by adding short polyols (1.2-propylene diol, ethylene glycol, glycerol, sorbitol, etc.) to the oscillatory mixture, which increases significantly the solubility of perborate due to the formation of perborate-polyol complex. We do not expect serious interference between the polyols and the main components BO${\;}_{3}^{-}$ and S_2_O${\;}_{3}^{2-}$ but if reduction of the Cu(II) catalyst by the polyol takes place, oscillations may not occur in the combined system. If our efforts are successful, phase diagrams in different parameter spaces will be established.

The non-linearity originates from the peroxo group present in both oxidants, therefore the set of reaction which produces the oscillatory dynamics may be partly similar or even identical in both ${\text{BO}}_{3}^{-}$ and H_2_O_2_ oscillators. In the taxonomy of the liquid phase chemical oscillators the systems based on the Cu(II)-catalyzed perborate oxidation of S_2_O${\;}_{3}^{2-}$ and SCN^−^ can be referred to as borate-assisted peroxide oscillators.

## Data Availability Statement

The original contributions presented in the study are included in the article/supplementary material, further inquiries can be directed to the corresponding authors.

## Author Contributions

KK-C, EP-T, and MO performed the experiments. IM and IS made the numerical simulations. KK-C, MO, and IS wrote the manuscript. MO supervised the whole project. All authors revised and checked the draft. All authors contributed to the article and approved the submitted version.

## Conflict of Interest

The reviewer JH declared a past co-authorship with one of the authors IS to the handling editor. The remaining authors declare that the research was conducted in the absence of any commercial or financial relationships that could be construed as a potential conflict of interest.
